# Ig Glycosylation in Ulcerative Colitis: It’s Time for New Biomarkers

**DOI:** 10.3389/fphar.2021.654319

**Published:** 2021-04-09

**Authors:** Riccardo Capecchi, Paola Migliorini, Federico Zanzi, Simona Maltinti, Ilaria Puxeddu, Nicola de Bortoli, Massimo Bellini, Francesco Costa, Santino Marchi, Lorenzo Bertani

**Affiliations:** ^1^ Department of Clinical and Experimental Medicine, University of Pisa, Pisa, Italy; ^2^ Department of Translational Research and New Technologies in Medicine and Surgery, University of Pisa, Pisa, Italy; ^3^ IBD Unit, Department of General Surgery and Gastroenterology, Pisa University Hospital, Pisa, Italy

**Keywords:** Ulcerative colitis, IBD, Biomarker, anti-TNF, glycosylation

## Abstract

**Background:** Ulcerative colitis (UC) is a chronic relapsing disease, which needs a continue monitoring, especially during biological therapies. An increasing number of patients is treated with anti-Tumor Necrosis factor (TNF) drugs, and current research is focalized to identify biomarkers able to monitor the disease and to predict therapeutic outcome.

**Methods:** We enrolled consecutive UC patients treated with anti-TNF, naïve to biologic drugs. Therapeutic outcome was evaluated after 54 weeks of treatment in terms of clinical remission (Partial Mayo Score -PMS- <2) and mucosal healing (Mayo Endoscopic Score <2). On serum samples collected at baseline and after 54 weeks of treatment, a Lectin-based ELISA assay was performed, and specific glycosylation patterns were evaluated by biotin-labelled lectins. We have also collected 21 healthy controls (NHS) samples, age and sex-matched.

**Results:** Out of 44 UC patients enrolled, 22 achieved clinical remission and mucosal healing after 54 weeks. At baseline, when Protein A was used as coating, UC patients non-responders showed a reduced reactivity to Jacalin (JAC) in comparison with NHS (*p* = 0.04). After one year of treatment, a decrease in JAC binding was seen only in responders, in comparison with baseline (*p* = 0.04). When JAC binding was tested selecting IgG by means of Fab anti-IgG Fab, UC patients displayed an increased reactivity after anti-TNF therapy (*p* < 0,0001 vs controls). At baseline, PMS inversely correlates with JAC binding when Fab anti-IgG Fab was used in solid phase (*r* ^2^ = 0,2211; *p* = 0,0033). Patients with higher PMS at baseline (PMS ≥5) presented lower binding capacity for JAC in comparison with NHS and with lower PMS patients (*p* = 0,0135 and *p* = 0,0089, respectively).

**Conclusion:** Ig glycosylation was correlated with clinical and endoscopic activity in patients with UC. JAC protein A-selected Ig showed a possible role in predicting therapeutic effectiveness. If these data would be confirmed, Ig glycosylation could be used as biomarker in UC.

## Introduction

Ulcerative colitis (UC) is a chronic relapsing disease, characterized by an inflammation affecting the colon and the rectum with superficial mucosal ulceration, rectal bleeding, diarrhea and abdominal pain (Ungaro et al., 2017). Tumor necrosis factor (TNF) plays an important role in UC pathogenesis. Indeed, several immune cells produce high levels of TNF, and this cytokine is known to mediate several pro-inflammatory functions in the inflamed mucosa, promoting even tissue injury (Neurath, 2014). The mechanisms behind the epithelial cell damage are only partially known, but an interesting paper by Nenci et al.(Nenci et al., 2007) showed how TNF induces intestinal cell apoptosis, or, better, “necroptosis”, by activating intracellular specific pathways. TNF activates a receptor-interacting protein kinase-1 (RIPK1), and when caspase-8 activity is suppressed, RIPK1 could promote necroptosis by interacting with receptor-interacting protein kinase-3, which regulates the phosphorylation of mixed lineage kinase domain-like protein (MLKL) (Zhou et al., 2020). This translocation of MLKL results to membrane perforation, and subsequently releases damage-associated molecular patterns into extracellular environment, triggering necroptosis (Royce et al., 2019). On this basis, the monoclonal antibodies anti-TNF, like infliximab (IFX) and adalimumab (ADA) were developed for treatment of UC.

Anti-TNF were demonstrated useful in inducing clinical remission, revolutionizing the management of UC in the past two decades, since their use shifted the treatment goals from symptom control to sustained corticosteroid-free remission, and, to a lesser extent, to mucosal healing (Ungaro et al., 2019). However, more than 30% of the UC patients receiving anti-TNF agents do not respond to treatment, and a significant proportion experience a loss of response or intolerance to treatment, even during the first year (Gisbert et al., 2015). An early prediction of therapeutic outcome is one of the most important challenge of clinicians to optimize therapeutic management.

Immunoglobulin (Ig) glycosylation has a strong impact on antibody function, by modifying protein conformation and IgG affinity for Fc-Receptors and complement (Jennewein and Alter, 2017). The conserved N-linked glycosylation site at the asparagine 297 (Asn297) in each of the Cγ2 regions of Fc fragment presents an arboreal glycan structure with two N-acetylglucosamine (GlcNAc) and three mannose (Man) residues, a “core” at which two additional GlcNAc groups are linked, forming the biantennary branches. Galactose (Gal) addition varily occur on each branch, permitting a potential terminal addition of a sialic acid. Only a minority of the chains are terminated by sialic acid (usually α2,6-linked) (Anthony et al., 2008). Fucose residue at the core GlcNAc can be present as well, alternatively substituted by bisecting GlcNAc (Epp et al., 2016). The peculiar position of the structure, near the cavity between the two IgG heavy chains, influences protein stability and Fc effector functions (Yamaguchi et al., 2006; Raju, 2008). Similar glycan residues are present on IgG Fab portions, modifying antigen recognition and binding (van de Bovenkamp et al., 2016).

Numerous immune-relates diseases, such as rheumatoid arthritis, anti-phospholipid syndrome, and ANCA-associated vasculitis among others, present peculiar modifications of glycan residues in circulating Ig (Parekh et al., 1988; Stümer et al., 2017; Culver et al., 2019). In particular, the majority of studies were conducted in rheumatoid artrithis, where anti-TNF treatment can modify peripheral IgG glycan spectrum (Pasek et al., 2006; Croce et al., 2007; Van Beneden et al., 2009). Recently, similar findings were also demonstrated in Inflammatory Bowel disease (IBD) (Trbojevic Akmacic et al., 2015; Simurina et al., 2018), posing the basis for further studies.

Several biomarkers have been proposed in order to evaluate disease activity and predict therapeutic response to anti-TNF in UC patients (Verstockt et al., 2019; Bertani et al., 2020a; Bertani et al., 2020c; Barberio B. et al., 2020; Bertani et al., 2021). However, the role of Ig glycosylation has never been studied with this purpose. To evaluate this hypothesis, we develop a lectin-ELISA assay in order to estimate an affinity spectrum for each lectin and correlate these results with clinical and endoscopic data.

## Materials and Methods

### Patients and Controls

We conducted a retrospective analysis of biological samples of patients with UC collected prospectively at IBD Unit of Pisa University Hospital from March 2018 to March 2020. The diagnosis of UC was previously confirmed by histology. According to statistical power plan, we chose to enroll 44 consecutive patients with UC treated for the first time with anti-TNF (IFX biosimilars CT-P13 or SB2, or ADA originator), divided in 22 responders and 22 non-responders. Therapeutic response was defined in terms of clinical remission (Partial Mayo Score -PMS- <2, without concomitant steroid therapy) and mucosal healing (Mayo Endoscopic Score <2) at week 54. All patients presented a moderate to severe disease activity at baseline (evaluated according to Mayo Score), and underwent a colonoscopy at baseline and after 54 weeks of treatment. We collected age, sex, smoke habits and disease extension at baseline for each patient. Moreover, C-reactive protein (CRP) and fecal calprotectin at baseline and after 54 weeks of treatment were collected as well. In case of non-response, anti-drug antibody development was evaluated by using specific ELISA kit (Theradiag®, Marne-la-Valle’e, France).

A sample of 9 ml of whole blood was drawn at baseline and after 54 weeks of treatment, immediately before drug administration. It was centrifuged at 4000 rpm, aliquoted and frozen at 20°C, according to our standard procedures for every patient treated with biologics at our Unit. Indeed, every patient treated with this drugs is monitored at our Unit with blood tests every drug infusion (in case of intravenous therapies) or every 8 weeks (in case of subcutaneous ones), and 9 ml of blood were used to create a Biobank of serum samples. Moreover, we have collected 21 healthy controls samples, age and sex-matched.

The present study was approved by the local Ethics Committee, and all patients gave their written informed consent for collection and publication of data.

### Lectin-Based ELISA

In order to detect specific glycan structures exposed on IgG, a Lectin-based ELISA assay was performed on the serum samples (Sjöwall et al., 2015; Stümer et al., 2017). Specific glycosylation patterns were evaluated by biotin-labelled lectins: aleuria aurantia lectin (AAL), lens culinaris agglutinin (LCA), O-glycosidically linked galactose/N-acetylgalactosamine (GalNAc) ligand jacalin (JAC) and sambucus nigra lectin (SNA) (Vector Laboratories, United States).

Protein A from *Staphylococcus aureus* (Sigma-Aldrich) was diluted in coating buffer (0.1 M Na_2_Co_3_/NaHCO_3_, pH 9,6) to 20 μg/ml and applied onto 96-well MaxiSorpTM microtitre plates (Nunc, Roskilde, Denmark; F96) at 4° overnight (50 µL/well). Alternatively, Fab2-fragment of goat anti-human Fab-specific IgG (Jackson Laboratories Immunoresearch, West Grove, PA, United States) was used at 2 μg/ml.

A blocking buffer (60 µL/well) was then applied onto the plates at room temperature (RT) for 1 h. As blocking buffer, we used 3% deglycosylated gelatin with 0.1% CaCl2 and 0.1% MgCl2. Gelatin was achieved by treatment with periodic acid for 24 h and subsequent dialysis against TBS-Ca-Mg until a pH -value of 7,4 was reached. Sera were diluted 1:500 in washing solution (TBS-Ca-Mg 0.05% Tween-20) and incubated at RT for 3 h (50 µL/well). After 3 washes with washing solution (150 µL/well), plates were incubated at RT for 1 h with biotin-labelled lectins diluted in TBS Ca-Mg (50 mcl/well), at various concentrations (AAL 100 ng/ml, LCA 100 ng/ml, GalNac -Jacalin 50 ng/ml, SNA 50 ng/ml). After washing, HRP-conjugated streptavidin in TBS Ca-Mg was incubated for ½ h at RT (50 µL/well), then washed again and Substrate Solution (TMB) was added (75 µL/well). After 10–15 min stopped with H_2_SO_4_ (37.5 mcl/well). Optical density values were obtained with the ELISA-reader employing a 450 nm filter. Each serum was tested in duplicate; the final value was obtained by the mean of the two results minus blank. Three samples were used as inter-assay controls. Each test was performed in blind, results were expressed as Optical Density (OD).

### Statistical Analysis

The non-parametric Mann–Whitney *U*-test was used to compare the patients’ group and the normal subjects. Spearman test was used for correlation analysis. Differences between groups were considered to be significant at a *p* value of <0.05. Statistical analyses were performed with GraphPad Prism 5.0 (GraphPad Software, Inc., San Diego, CA, United States).

## Results

Clinical and serological features of the enrolled patients are summarized in Table 1. Nineteen out of 44 were male, with a mean age of 31 years. Asthma was the most common comorbidity reported (3 out of 44). Five patients were smokers. Only two patients presented isolated proctitis, while 14 had pancolitis. Before anti-TNF therapy, but not at baseline, seven and 27 patients were treated with topical or oral steroid, respectively (mean dose 15 mg/die prednisone equivalent, SD ± 11). Median CRP level at baseline were 0.65 mg/dl (IQR 0.3–1.38), with 18 patients presenting CRP over normal value (0.5 mg/dl). Median fecal calprotectin was 256 mg/kg (IQR 71–476). Over the 22 patients enrolled as non-responders, seven patients presented anti-drug antibodies at treatment discontinuation (at week 54).

**TABLE 1:** Demographic, clinical and serologic characteristics of patients’ cohort.

	UC patients (*n = 44*) *Baseline*	*Follow-up*		
Age (years) (mean and IQR)	31 (24–45)			
Sex (M/F)	19/25			
CRP (mg/dl)	0.65 (0.3–1.38)	0.3 (0.2–0.63)	p = 0.06
Fecal calprotectin (mg/kg)	266.0 (83–464)	126 (50.5–504.0)	p= 0.29
MES	3 (2–3)	2 (1–3)	p = 0.0002
PMS	4 (2–5)	2 (0–5)	*P* = 0.13
AAL (PrA, OD)	0.404 (0.296–0.603)	0.425 (0.297–0.561)	p = 0.19
LCA (PrA, OD)	0.74 (0.579–0.824)	0.741 (0.59–0.886)	p = 0.49
SNA (PrA, OD)	0.587 (0.558–0.63)	0.594 (0.565–0.624)	p = 0.8
JAC (PrA, OD)	0.696 (0.556–0.781)	0.635 (0.536–0.737)	p= 0.07
JAC (fab anti-Fab, OD)	0.252 (0.184–0.5)	0.692 (0.372–0.943)	p < 0.0001

Data are expressed as median (interquartile range–IQR).

*CRP* C-reactive protein, *MES* Mayo Endoscopic Score, *PMS* Partial Mayo Score, *AAL* Aleuria Aurantia Lectin, *LCA* Lens Culinaris Agglutinin, *SNA* Sambucus Nigra Lectin, *JAC* Jacalin, *PrA* Protein A, *Fab anti-Fab* Fab anti-IgG Fab, *OD* Optical Density.

Reactivity for AAL, LCA and SNA showed no differences between healthy controls and UC patients at baseline nor after therapy (Table 1). IFX (both biosimilars) and ADA presented negligible lectin binding in comparison with healthy subjects (NHS) and patients (data not shown).

**FIGURE 1:**
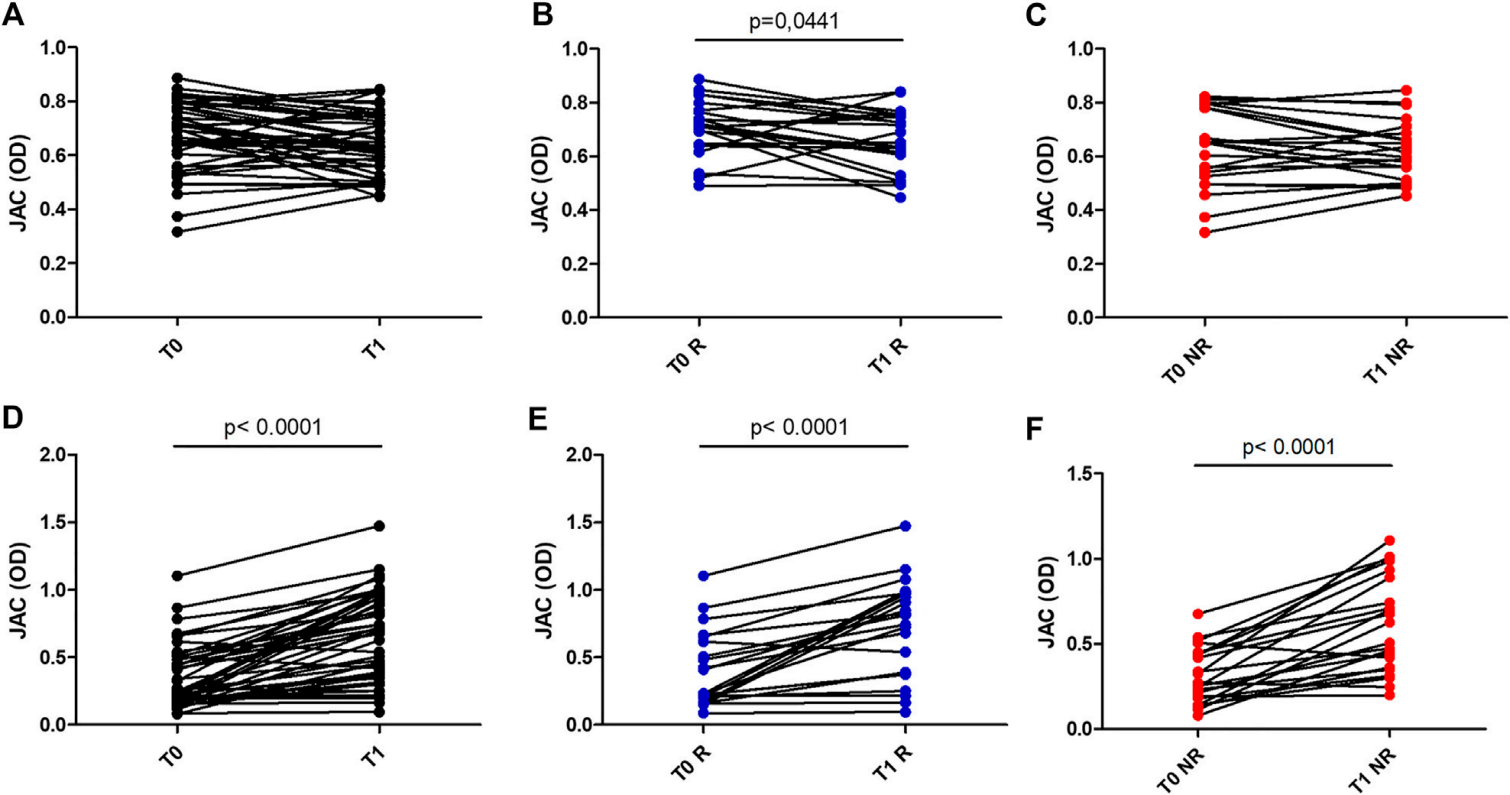
JAC affinity for IgG complex at baseline (T0) and after anti-TNF therapy (T1), expressed as Optical Density (OD). A,B,C: coating with Protein A in all patients **(A)**, in Responders **(B)** and Non Responders **(C)**. D,E,F: coating with Fab anti Fab IgG in all patients **(D)**, in Responders **(E)** and Non Responders **(F)**. All Responders (R) dots were in blue, All Non Responders (NR) dots were in red.

At baseline, when Protein A is used as coating, sera from UC patients non responders to anti-TNF therapy showed a reduced reactivity to JAC in comparison with healthy controls (*p* = 0.04) (Figure 1). After one year of treatment, a modest but significant decrease in JAC binding was seen only in cohort of UC patients responders to therapy, in comparison with baseline (*p* = 0.04, Wilcoxon paired test).

**FIGURE 2:**
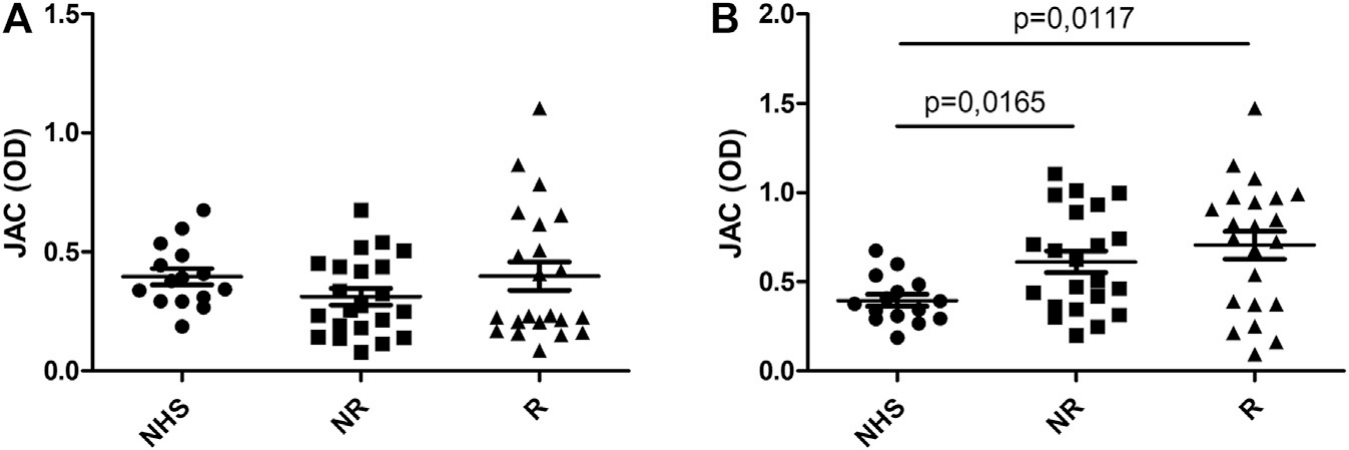
JAC affinity for IgG complex in Ulcerative Colitis compared to healthy subjects (NHS). Lectin-based ELISA coated with Fab anti Fab IgG. **(A)**: at baseline (T0); **(B)**: after anti-TNF therapy (T1). NHS = healthy subjects, R = responders, NR = non responders. All values are expressed as Optical Density (OD).

Conversely, when JAC binding was tested selecting IgG by means of Fab anti-IgG Fab, UC patients display an increased reactivity after anti-TNF therapy (*p* < 0,0001 vs. controls) (Figure 2). Such an increase was higher in responders vs. non-responders (*p* = 0,0086 and *p* = 0,0047, respectively).

No correlation was detected with regard for patients’ age, sex, smoke habits, steroid use, baseline or follow-up CRP and fecal calprotectin levels (data not shown). No differences were highlighted between patients stratified according to Mayo Endoscopic Score before or after therapy.

**FIGURE 3:**
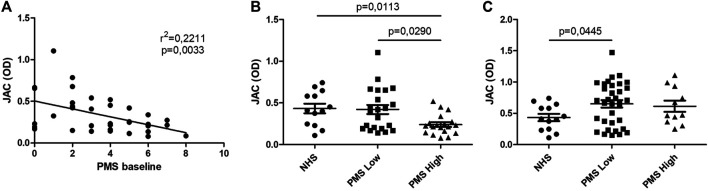
JAC affinity for IgG complex in comparison with Partial Mayo Score (PMS). Lectin-based ELISA coated with Fab anti-IgG Fab. **(A)**: linear correlation between PMS and JAC affinity. **(B, C)**: JAC affinity for IgG complex in Ulcerative Colitis with Low PMS (≤5) and High PMS (>5) compared to healthy subjects (NHS), at baseline **(B)** and after anti-TNF therapy **(C)**. All values are expressed as Optical Density (OD).

At baseline, PMS inversely correlates with JAC binding when Fab anti-IgG Fab is used in solid phase (*r* ^2^ = 0,2211; *p* = 0,0033), while statistical significance was lost when evaluated in follow-up samples (Figure 3). Patients with higher PMS at baseline (PMS ≥5) presented lower binding capacity for JAC in comparison with NHS and with lower PMS patients (*p* = 0,0135 and *p* = 0,0089, respectively). At week 54, no differences were seen between high PMS and low PMS patients in terms of JAC reactivity (Figure 3).

## Discussion

The present study showed that Ig glycosylation could be used as biomarker of disease activity in UC, especially before starting a biological therapy with anti-TNF.

UC is a chronic inflammatory disease which needs a continue monitoring. In this perspective, the use of biomarkers is crucial, since they could allow a more detailed evaluation of patients’ clinical conditions. Moreover, biomarkers able to reflect endoscopic activity are useful in reducing the number of endoscopic examinations, which are not always well accepted by patients. CRP is one of the most used biomarkers in inflammatory conditions, but its levels are not often correlated to UC activity, unlike Crohn’s disease (Saverymuttu et al., 1986). Conversely, fecal calprotectin is the most used biomarker in UC setting (Mumolo et al., 2018): it is reliable both in monitoring disease activity (Mumolo et al., 2018) and in predicting therapeutic effectiveness to anti-TNF drugs (Bertani et al., 2020a), due to its important correlation with endoscopic and histologic activity (D'Amico et al., 2020). However, fecal calprotectin levels could vary day-to-day, and several factors could modify them, such as the use of non-steroidal anti-inflammatory drugs or the presence of other inflammatory conditions (Bertani et al., 2020b). Therefore, several different serum biomarkers have been proposed, and the levels of Leucine-rich α-2 glycoprotein have demonstrated an interesting correlation with tissue inflammation in UC (Serada et al., 2012).

IgG glycosylation represent a post-translational modification capable of fine-tuning the antibody-mediated immune response. Many autoimmune conditions are associated with qualitative perturbations in circulating antibodies. Agalactosylation of N-linked glycans in IgG is considered a marker of inflammatory condition, as shown in patients with rheumatoid arthritis and systemic lupus erythematous (Parekh et al., 1988; Vuckovic et al., 2015). Biantennary agalactosylated antibodies, especially anti-citrullinated protein antibodies, are increased before the disease onset and are capable to interact with FcγRIIa, induce macrophage activation and, subsequently, production of Tumor Necrosis Factor α and IL6 (Clavel et al., 2008).

In their seminal work in 2006, Kaneko and Nimmerjahn demonstrated an anti-inflammatory activity of IgG, as a result of Fc sialylation (Kaneko et al., 2006). This modulation required DC-SIGN receptor interaction (Yabe et al., 2010). Since galactosylation is mandatory for subsequent sialylation, some authors suggested that a lack of galactose induced inflammation reducing sialic immunomodulant activity. Recently, Pfeifle et al.(Pfeifle et al., 2017) demonstrated that IL23 specifically suppress ST6GAL1 expression in plasma cells, inducing a phenotypic switch in Th17 compartment in an IL22/IL23-dependent manner. In turn, ST6GAL1 downregulation in plasma cells lead to asialylated antibody production, shifting toward a pro-inflammatory antibody repertoire (Pfeifle et al., 2017).

A genome-wide association study of IgG N-glycosylation showed that variants affecting the expression of genes involved in the regulation of glycoenzymes colocalize with variants affecting risk for inflammatory diseases: notably, authors observed pleiotropy between variants in the IKZF3-ORMDL3-GSDMB-ZPBP2 locus with inflammatory conditions, including UC (Klaric et al., 2020). An international research group coordinated by prof Lauc clearly described in two recent papers specific modifications in Ig glycome of patients with IBD, analyzed by ultra-performance liquid chromatography (Trbojevic Akmacic et al., 2015) and mass spectrometry (Simurina et al., 2018). IBD patients presented lower levels of IgG galactosylation than controls, while a significant decrease in the proportion of sialylated structures was seen only in patients with Crohn’s disease. Notably, a decreased galactosylation was associated with more severe disease for all patients and with a major need for surgery.

Several studies showed that agalactosylation (indicated also as IgG-G0) could be reverted in therapeutic conditions capable of ameliorating the inflammatory diseases. In rheumatoid arthritis, steroid and anti-TNF therapy induce an increase IgG sialylation (Pasek et al., 2006; Croce et al., 2007; Van Beneden et al., 2009). To our knowledge, this is the first attempt to evaluate IgG glycan patterns in UC patients before and after anti-TNF therapy.

We implemented a Lectin-based ELISA, modifying protocols already published (Sjöwall et al., 2015; Stümer et al., 2017). In order to explore IgG glycans, biotin-labelled lectins were used: AAL, that binds preferentially fucosylated residues; LCA, able to recognize tri-mannose N-glycan core; JAC, an O-glycosidically linked GalNAc ligand; and SNA, capable to bind sialylated residues. This test presents some advantages in comparison with mass spectrometry or capillary electrophoresis: it is affordable, easy to realize in a lab, and did not need denaturation treatments for sample analysis. However, it presents some limits. First, lectins present a gradient of binding capacity for many glycan residues: in this way, a specific lectin reactivity for IgG-complex should be carefully interpreted as the resultant of many different interactions between the lectin and glycan structures, leading to a less specific result in comparison with other technologies. Second, the system is not able to distinguish between Fab and Fc glycosylation, nor can estimate the percentage of biantennary or monoantennary residues, but evaluate IgG-complex as a whole. Moreover, some authors stressed that a lectin-based ELISA could evaluate only IgG complex, e.g., circulating immunocomplex or IgG complexed with acute phase reactants such as CRP, or complement components such as C1q or C3c, since in physiological conditions these complexes are represent verily in peripheral blood (Sjöwall et al., 2015; Stümer et al., 2017). In our cohort of patients, CPR levels did not correlate with lectin binding; on the other hand, an old paper reported the presence of circulating immunocomplexes in UC, albeit clinical implications of this finding were dubious (Kemler and Alpert, 1980). However, a test more adherent to a physiological native IgG condition could be more informative in terms of clinical correlation in comparison with more sophisticated analyses.

Our results diverge when different type of coating was implemented. The putative role of JAC in predicting therapeutic effectiveness was not clearly demonstrated: it could be used as a biomarker of therapeutic response when we evaluated Protein A-selected Ig, whereas in Fab anti-IgG Fab ELISA it was not associated to therapeutic outcome.

The apparent contradiction between data obtained with Protein A or Fab anti-IgG Fab could be explained because of the different orientation of IgG-complexes on the plate. In fact, Fab anti-IgG Fab could expose more clearly the Fc fragment for lectin interaction, or could interfere with immunocomplex formation. Moreover, Protein A (usually used in IgG purification process) could hypothetically recognize with low affinity also circulating IgA. JAC presented high affinity for O-linked GalNAc, which are a minority in human IgG, mainly limited to IgG3 subclass, while classically represented in IgA (Plomp et al., 2015). When IgG selection is more specific, as in the case of Fab anti-IgG Fab, the use of JAC could lead a more informative result. In fact, it has been shown that JAC can recognize as well lactose and galactose, but also mannose and oligomannosides (Bourne et al., 2002). The correlation with a clinical composite score, such as PMS as demonstrated by our results, strongly supports this conclusion. From this point of view, what JAC shows in UC patients after anti-TNF treatment is in line with similar modifications already described in rheumatoid arthritis (Pasek et al., 2006; Croce et al., 2007; Van Beneden et al., 2009; Stümer et al., 2017), suggesting a not disease-specific pathway of anti-TNF in restoring fully glycosylated moiety. Further experiments with mass spectrometry are needed to confirm these findings.

It is worthy to mention that seven patients non-responders developed anti-drug antibodies. This peculiar subgroup did not present a specific glycosylation pattern in comparison with other UC patients. In this perspective, the putative correlation between JAC and clinical activity could be reliable even in case of development of anti-drug antibodies, if confirmed in larger samples.

The present study has some limitations. The retrospective analysis undoubtedly limited the significance of our results. However, it is worth to note that the biological samples and clinical and endoscopic data were collected prospectively, and this procedure limited the possible biases of a typical retrospective study of medical records. Other important limitations are related to the relatively small sample size and to the lack of the evaluation of serum drug levels and anti-drug antibodies in all patients. We should highlight how the present study should be intended as a pilot, explorative study, and we have performed drug levels and antibodies according to the current guidelines (Papamichael et al., 2019), only in case of non-response. A larger cohort would probably increase the significance of our results, in particular with regard for the correlation with endoscopic activity at baseline, where only a trend was showed by our results. Furthermore, larger studies are needed to better characterize the putative role of JAC as a biomarker of therapeutic response to anti-TNF.

Conversely, the present study has an important strength: this is the first time when the role of Ig glycosylation was showed in a cohort of patients with UC all treated with anti-TNF for the first time, highlighting the differences with NHS. Furthermore, the correlation with clinical and endoscopic (although not significant) activity, as well as the behavior during anti-TNF treatment is in line with results obtained with mass spectrometry in patients with IBD and similar to the findings in other immune-related diseases. Therefore, our results pave the way for future studies aimed at clarifying the possible use of Ig glycosylation (particularly JAC), in UC setting.

To conclude, Ig glycosylation may be correlated with clinical and endoscopic activity in patients with UC. Moreover, when a specific Fab anti-IgG Fab was used, JAC affinity increased after anti-TNF therapy and inversely correlated with clinical activity. JAC protein A-selected Ig showed a possible role in evaluating therapeutic effectiveness. Our JAC-labelled ELISA could be an affordable, feasible and reproducible test to evaluate the affinity spectrum of native circulating Ig-complex and it could be a candidate as a new biomarker in UC, if confirmed in larger cohorts.

## Data Availability

The original contributions presented in the study are included in the article/Supplementary Material, further inquiries can be directed to the corresponding author.
